# Associations between respiratory function, balance, postural control, and fatigue in persons with multiple sclerosis: an observational study

**DOI:** 10.3389/fpubh.2024.1332417

**Published:** 2024-03-20

**Authors:** Rodrigo Sanchez-Ruiz, Marta de la Plaza San Frutos, M. Dolores Sosa-Reina, Ismael Sanz-Esteban, Maria García-Arrabé, Cecilia Estrada-Barranco

**Affiliations:** Faculty of Physical Activity and Sport Sciences, European University of Madrid, Madrid, Spain

**Keywords:** multiple sclerosis, fatigue, postural control, equilibrium, respiratory function

## Abstract

**Introduction:**

Fatigue, postural control impairments, and reduced respiratory capacities are common symptoms in persons diagnosed with Multiple Sclerosis (MS). However, there is a paucity of evidence establishing correlations among these factors. The aim of this study is to analyze respiratory function in persons with MS compared to the control group as well as to analyze the relationship between fatigue, respiratory function and postural control in persons with MS.

**Materials and methods:**

A total of 17 persons with MS and 17 healthy individuals were enrolled for this cross-sectional study. The evaluated parameters included fatigue assessed using the Visual Analog Scale-fatigue (VAS-F) and the Borg Dyspnea Scale, postural control assessed through the Mini Balance Evaluation System Test (Mini-BESTest), Berg Balance Scale (BBS), Timed Up and Go (TUG) test, and Trunk Impairment Scale (TIS); and respiratory capacities measured by Maximum Inspiratory Pressure (MIP), Maximum Expiratory Pressure (MEP), Forced Vital Capacity (FVC), Forced Expiratory Volume in the first second (FEV1), FEV1/FVC ratio, Diaphragmatic excursion and diaphragmatic thickness.

**Results:**

A very high correlation was observed between the Borg Dyspnoea Scale and the BBS (*r* = −0.768), TUG (0.867), and Mini-BESTest (*r* = −0.775). The VAS-F exhibited an almost perfect correlation solely with the TUG (0.927). However, none of the variables related to fatigue exhibited any correlation with the respiratory variables under study. Balance-related variables such as BBS and Mini-BESTest demonstrated a very high and high correlation. Respectively, with respiratory function variables MEP (*r* = 0.783; *r* = 0.686), FVC (*r* = 0.709; *r* = 0.596), FEV1 (*r* = 0.615; *r* = 0.518). BBS exhibited a high correlation with diaphragmatic excursion (*r* = 0.591). Statistically significant differences were noted between the persons with MS group and the control group in all respiratory and ultrasound parameters except for diaphragmatic thickness.

**Conclusion:**

The findings suggest that decreased postural control and balance are associated with both respiratory capacity impairments and the presence of fatigue in persons with MS. However, it is important to note that the alterations in respiratory capacities and fatigue are not mutually related, as indicated by the data obtained in this study. Discrepancies were identified in abdominal wall thickness, diaphragmatic excursion, and respiratory capacities between persons with MS and their healthy counterparts.

## 1 Introduction

Multiple Sclerosis (MS) represents a clinically intricate entity, characterized by its chronic, progressive, idiopathic nature with an inflammatory and autoimmune profile inducing demyelination in the central nervous system (1, 2), with a global incidence affecting 1.3 million individuals. MS exhibits a significantly higher prevalence in women, with a ratio of 3:1 (3). The symptomatic and evolutionary diversity of MS is a distinctive facet of this pathology, demyelinating plaques, fundamental markers of the disease, manifest more frequently in areas such as the optic nerves, brainstem, spinal cord, and periventricular white matter (4). Characteristic functional deficits encompass a broad range of manifestations, including muscle weakness, sensory disturbances, reduced passive mobility, spasticity, loss of postural control, pain, visual impairment, Ataxia, fatigue, incontinence, cognitive complications, and depression (5). Between 50 and 80 % of individuals affected experience disorders related to postural control and balance (6–8). Impaired breathing, postural control, and fatigue are also frequently reported (9).

Postural control, which refers to the body’s capacity to predict and respond to destabilizing forces, entails coordinated joint adjustments through synergistic sensorimotor processes (visual, Vestibular and proprioceptive) as well as activation of core musculature, encompassing the diaphragm, transverse abdominis, multifidus and pelvic floor (10, 11). Detecting and analyzing postural control deficits in persons with MS can contribute to a better understanding of balance impairments and facilitate the design of interventions aimed at reducing the risk of falls (12). Studies have recently delved into the interconnectivity between respiratory function and postural control. The term “CORE,” is often likened to a cylinder that surrounds vital organs. It is composed of abdominal muscles (13), lumbar muscles, pelvic muscles, gluteal muscles, the diaphragm above the cylinder, and the pelvic floor muscles that form its base (14–16). The CORE plays a fundamental role in postural control and constitutes an anatomical and functional system that serves as the center for the kinetic chains involved in all bodily movements. It is influenced by changes in intra-abdominal pressure orchestrated by diaphragmatic contraction, which gives the diaphragm an important role in trunk stability (17–19). During inhalation, it increases intra-abdominal pressure, thereby enhancing stability (20). In summary, the functions of stabilization and respiration are neuro-muscularly interdependent.

Several authors have examined this relationship in various pathologies. Massery et al. (21) emphasized the significance of glottic control in maintaining intra-abdominal pressure. Studies have also demonstrated that changes in postural control can impact respiratory function in different neurological diseases. Yoon et al. (22) established that core musculature training could enhance diaphragmatic thickness and excursion. Lee et al. (23) demonstrated the impact of respiratory training on balance and postural control in stroke patients (21, 22). The strong correlation between respiratory function and postural control has also been evident in Parkinson’s disease patients (24). Conversely, abnormal breathing patterns and reduced respiratory capacities could influence postural control. Even in healthy subjects, a relationship between respiratory patterns and balance has been identified (18).

Fatigue is a common symptom experienced by persons with MS (25, 26) with two distinct categories: central fatigue, caused by changes in the central nervous system, and peripheral fatigue, which is related to alterations in the neuromuscular function (27). Research indicates that there may be a relationship between fatigue and structural changes in the nervous system, such as subcortical atrophy and impairment of basal ganglia circuits (25, 28, 29). Furthermore. previous studies have identified a correlation between respiratory muscle strength, functionality and fatigue (30–32). However, to our knowledge, no study concurrently examines the relationship between fatigue and balance and respiratory function in persons with MS.

The aim of this study is to investigate changes in the respiratory function of persons with MS. To this end, we compared diaphragmatic thickness, diaphragmatic excursion, peak inspiratory pressure (MIP), peak expiratory pressure (MEP), forced vital capacity (FVC), forced expiratory volume in the first second (FEV1) and its coefficient (FEV1/FVC); variables in these persons with those of healthy subjects. In addition, we aim to analyze the associations between respiratory function, diaphragmatic and muscular morphology, postural control, balance and fatigue in persons with MS. We believe that a deeper understanding of these relationships could provide valuable information for selecting more effective therapeutic approaches to treat MS.

## 2 Methods

A quantitative, cross-sectional, observational, and analytical study was conducted, incorporating a control group. The data collection process adhered to the STROBE (STrengthening the Reporting of OBservational studies in Epidemiology) (33), guidelines to standardize information gathering. Ethical approval was obtained from the Clinical Hospital San Carlos under the code 23/233-E_TFM.

Participants from the MS group were recruited from the MS Foundation of Madrid (FEMM) during March and April 2023. The inclusion criteria for this sample were persons diagnosed with MS, exhibiting a score < 5 on the Functional Ambulation Category (FAC), possessing adequate cognitive abilities for testing, and not exhibiting any other neurological, cardiorespiratory, or other impairments that could possibly interfere with the assessment. The control group participants were recruited via advertisements, striving for the same age, sex, weight, and height as the MS group subjects to ensure consistency. Individuals who have pathologies affecting the neurological, cardiac, respiratory, autoimmune, rheumatic, or traumatic systems that could impact testing results were omitted from the study.

The sample size was calculated at 15 participants per group using GRANMO^©^ software, accepting an alpha risk of 0.05 and a beta risk of 0.2 in a bilateral test, with an estimated 10% follow-up loss. In addition to sociodemographic variables, the study collected the following variables for each group.

To assess balance and postural control in both ambulatory individuals and persons with MS without trunk control, a battery of tests was administered: The Mini Balance Evaluation Systems Test (Mini-BESTest), a condensed version of the ‘Balance Evaluation System Test’ (34) comprising 14 items, was utilized to measure balance and postural control during both static and dynamic conditions. Each item is assigned three possible scores ranging from 0 to 2, with higher scores indicative of better balance. Notably, the Mini-BESTest exhibited high validity and specificity in persons with MS when compared to the Berg Balance Scale (BBS) (35). The Trunk Impairment Scale (TIS), designed to evaluate static and dynamic trunk stability in a seated position without back support, consists of 17 items scored according to a validated rubric. Higher scores on this scale signify improved balance, with a maximum achievable score of 23. The TIS has been validated for persons with MS (36). The BBS was employed to measure balance in a broad manner including postural changes, comprising 14 items. Each item was scored on a scale from 0 to 4, with higher scores denoting better balance. The maximum total score achievable on the BBS was 56 (37).

The Timed Up and Go (TUG) test was administered to assess functional mobility, including sit-to-stand transfers, walking and turning. Fatigue was assessed using the following scales: the Visual Analog Scale (VAS-F) for perceived fatigue, validated for use in persons with MS, measured daily perceived fatigue on a 10-point scale in response to the question “How much influence does fatigue have on your daily life (every life at home and at work) and on your relationships.” Subjects marked their response on a 10 cm line without numerical labels. This scale demonstrated construct validity when compared to the Modified Fatigue Impact Scale (MFIS) and the Fatigue Severity Scale (FSS) (38). The Modified Borg Dyspnea Scale was administered post the TUG to assess perceived dyspnea or fatigue after the test. Subjects assigned a number from 1 to 10 to indicate the level of dyspnea perceived immediately after test completion (39). This scale allowed for numerical quantification of perceived fatigue.

Respiratory muscle strength was assessed through measurements of maximal inspiratory pressure (MIP) and maximal expiratory pressure (MEP). Following FVC and FEV1 measurements, participants rest for 5 min, using the same position and nose clip, the examiner demonstrates and oversees maneuvers. Starting with Maximal Inspiratory Pressure (MIP), participants perform six repetitions, followed by Maximal Expiratory Pressure (MEP) maneuvers, one-minute rests separate each. The highest MIP and MEP values (cm3 H20) are compared with reference values (40). Respiratory function will be assessed using spirometric tests following the SEPAR Guidelines (2013) (41). An open-circuit pneumotachograph or spirometer will be employed. Measurements include FVC, indicating the maximum volume of air, in milliliters, a participant can inhale during forced inspiration. Additionally, the maximum expiratory volume in the first second (FEV1), measured in milliliters, will be recorded. Providing insights into pulmonary elastic quality, the morphological evaluation of the diaphragm was performed by ultrasound assessment adhering to the guidelines established by the RUSI (Review of Ultrasound in Immobility Syndrome) (42). The measurements were taken by a single evaluator who was unaware to which group each subject belonged to and was trained in the ultrasound technique, a high-quality ultrasound system (LOGIQ V2; GE Healthcare, United Kingdom). Below, we provide a comprehensive description of the procedures for measuring diaphragmatic thickness and excursion.

For the assessment of diaphragmatic thickness, we utilize a linear probe with a frequency range of 10–12 MHz. Persons are comfortably positioned in the supine posture, and the examination focuses on the anterior aspect of the right hemidiaphragm. Using the hepatic window as our point of access, the ultrasound probe is positioned perpendicularly within the intercostal space between the ninth and tenth ribs, aligned with the axillary line. Within the ultrasound image, we identify three distinct parallel layers, each with varying echogenicity corresponding to the pleura, diaphragm, and peritoneum. To measure diaphragmatic thickness, we employ the M-mode, freezing the image during an unforced expiration. Three separate measurements are taken during each examination. and the final result is derived from the mean of these three measurements (43, 44).

For the assessment of diaphragmatic excursion, we use a convex ultrasound probe with a frequency range of 2.5 to 3.5 MHz. Persons are placed in the supine position with a 45° headrest angle and are encouraged to rest quietly with closed eyes before the examination. The ultrasound probe is positioned beneath the right costal arch, along a line corresponding to the midpoint of the clavicle and oriented cranially. This positioning allows us to visualize the dome of the right hemidiaphragm as a prominent hyperechoic line. To measure diaphragmatic excursion, we record measurements for both normal and forced breathing. Conducting this process three times, the measurement is taken from the highest point of the sinuous curve of the diaphragmatic dome to the lowest point it reaches during inspiratory contraction. The final result is calculated as the mean of the three measurements. Standardization of these procedures guarantees consistency in measurement and results across our study (43, 44).

Statistical analysis included Spearman correlation to explore relationships between balance, fatigue, and respiratory variables in persons with MS. Additionally, correlation analysis between fatigue and respiratory or balance variables was conducted. Following Hopkins et al. (45), the following levels of correlation coefficient were established: non-existent (*r* < 0.1); Low (*r* = ≥0.1 < 0.3); Moderate (*r* = ≥0.3 < 0.5); high (*r* = ≥0.5 < 0.7); very high (*r* = ≥0.7 < 0.9) and almost perfect (*r* ≥ 0.9).

To compare persons with MS and healthy individuals, the Mann–Whitney U test was employed. A statistical significance level of *p* = 0.05 was set.

## 3 Results

A total of 34 subjects were recruited. Seventeen persons with MS were selected from the FEMM and assessed to ensure they met the inclusion criteria. Similarly, 17 healthy subjects who met the inclusion criteria were chosen for the control group. No statistically significant differences were observed in the sociodemographic variables studied between groups. The characteristics of the sample are presented in Table 1.

**Table 1:** Sociodemographic characteristics of the participants.

	MSG *n* = 17	CG *n* = 17
Sex (n)	4 M; 13 W	4 M; 13 W
Age (years)	53 (47. 67)	55 (51. 61)
Weight (kg)	72 (60. 77)	69 (52. 74)
Height (cm)	168 (162. 174)	168 (160. 169)
Type of ME (n)	8 P–P; 5 S-P; 4 R-R	

Data are presented in absolute frequency or in median (25th percentile; 75th percentile). MSG, Multiple Sclerosis Group; GC, Control Group; MS, Multiple Sclerosis; M, Men; M, Women; P–P, Primary Progressive; S-P, Secondary Progressive; R-R, Relapsing–Remitting. *p < 0.05 between groups.

### 3.1 Differences between MS group and healthy controls

Although it is common for healthy individuals to show no fatigue or balance problems, we refrained from directly comparing these variables between healthy individuals and those with MS, as these variables have been previously reported. The results show statistically significant differences between the two groups in all the variables studied, except for relation FEV1/FVC and diaphragmatic thickness (Table 2).

**Table 2:** Differences between MS group and healthy controls.

	MSG (*n* = 17)	CG (*n* = 17)	*p* value
VAS-F	6 (8; 2)	1 (1; 0)	**<0.001**
Borg	0 (3; 0)	0 (1; 0)	**<0.001**
MIP	57 (41; 42)	78 (57; 90)	**0.001**
MEP	77 (50; 56)	99 (77; 115)	**<0.001**
% FVC	79 (64; 70)	92 (79; 110)	**0.003**
% FEV1	84 (66; 70)	96 (84; 112)	**0.002**
%FEV1/FVC	82 (73; 88)	80 (77; 84)	0.666
DE	5.51 (4.14; 3.95)	6.14 (5.51; 7.04)	**0.01**
DT	0.17 (0.18; 0.18)	0.21 (0.17; 0.27)	0.147

Data are presented in median (25th percentile; 75th percentile). MSG, Multiple Sclerosis Group; GC, Control Group; MS, Multiple Sclerosis; MIP, Maximal Inspiratory Pressure; MEP, Maximal Expiratory Pressure; FVC, Forced Vital Capacity; FEV1, Forced Expiratory Volume in 1 s; DE, Diaphragmatic Excursion; DT, Diaphragmatic Thickness. Bold type in tables indicates statistical significance.

### 3.2 Internal correlation between measures instruments to evaluate fatigue, balance y respiratory outcomes

In the MS group, significant correlations were observed among most fatigue and balance assessment instruments, except for the TIS and TUG. Conversely, in the control group, only a correlation between the Mini-Bestest and BBS was found. Regarding respiratory variables, correlations were present among all variables in the MS group, except for FEV1/FVC and DT. In contrast, in the control group, a correlation was only observed between FVC and FEV1 (Table 3).

**Table 3:** Internal correlation between measures instruments to evaluate fatigue, balance y respiratory outcomes.

		Fatigue	Balance			Respiratory outcomes					
		VAS-F r (*p*)	TUG r (*p*)	BBS r (*p*)	MiniBESTest r (*p*)	MIP r (*p*)	MEP r (*p*)	FVC r (*p*)	FEV1 r (*p*)	FEV1/FVC r (*p*)	DE r (*p*)
Borg	MSG	0.818 **(*p* = 0.025)**									
	CG	0.188 (*p* = 0.470)									
	Combined	0.41 **(*p* = 0.04)**									
BBS	MSG		0.867 **(*p* = 0.012)**								
	CG		−0.397 (*p* = 0.115)								
	Combined		0.779 (*p* **< 0.001**)								
MiniBESTest	MSG		−0.883 **(*p* = 0.008)**	0.868 **(*p* < 0.001)**							
	CG		−0.471 (*p* = 0.057)	0.882 **(*p* < 0.001)**							
	Combined		−0.799 (*p* **< 0.001**)	0.973 (*p* **< 0.001**)							
TIS	MSG		−0.630 (*p* = 0.129)	0.972 **(*p* < 0.001)**	0.874 **(*p* = <0.001)**						
MEP	MSG					0.723 **(*p* < 0.001)**					
	CG					0.389 (*p* = 0.123)					
	Combined					0.674 **(*p* < 0.001**)					
FVC	MSG					0.858 **(*p* < 0.001)**	0.874 **(*p* < 0.001)**				
	CG					0.221 (*p* = 0.370)	0.132 (*p* = 0.612)				
	Combined					0.647 (*p* **< 0.001**)	0.641 (*p* **< 0.001**)				
FEV1	MSG					0.816 **(*p* < 0.001)**	0.840 **(*p* < 0.001)**	0.931 **(*p* < 0.001)**			
	CG					0.232 (*p* = 0.370)	0.194 (*p* = 0.456)	0.975 **(*p* < 0.001)**			
	Combined					0.629 (*p* **< 0.001**)	0.672 (*p* **< 0.001**)	0.969 (*p* **< 0.001**)			
FEV1/FVC	MSG					0.009 (*p* = 0.974)	0.229 (*p* = 0.376)	−0.179 (*p* = 0.492)	0.012 (*p* = 0.963)		
	CG					0.239 (*p* = 0.355)	0.049 (*p* = 0.852)	−0.252 (*p* = 0.328)	−0.194 (*p* = 0.456)		
	Combined					0.006 (*p* = 0.974)	−0.107 (*p* = 0.545)	−0.188 (*p* = 0.288)	−0.084 (*p* = 0.638)		
DE	MSG					0.484 **(*p* = 0.049)**	0.787 **(*p* < 0.001)**	0.722 **(*p* = 0.001)**	0.654 **(*p* = 0.004)**	−0.519 **(*p* = 0.033)**	
	CG					0.285 (*p* = 0.268)	0.327 (*p* = 0.200)	0.191 (*p* = 0.462)	0.326 (*p* = 0.202)	0.321 (*p* = 0.209)	
	Combined					0.507 **(*p* = 0.002)**	0.652 (*p* **< 0.001**)	0.542 (*p* **< 0.001**)	0.559 (*p* **< 0.001**)	−0189 (*p* = 0.284)	
DT	MSG					0.089 (*p* = 0.734)	0.129 (*p* = 0.621)	−0.043 (*p* = 0.869)	0.096 (*p* = 0.713)	0.069 (*p* = 0.792)	−0.075 (*p* = 0.773)
	CG					0.025 (*p* = 0.924)	0.009 (*p* = 0.924)	−0.386 (*p* = 0.126)	−0.330 (*p* = 0.195)	0.446 (*p* = 0.073)	0.417 (*p* = 0.095)
	Combined					0.221 (*p* = 0.208)	0.262 (*p* = 0.134)	−0.034 (*p* = 0.851)	0.033 (*p* = 0.852)	0.203 (*p* = 0.250)	0.312 (*p* = 0.073)

MSG, Multiple Sclerosis Group; CG, Control group; MIP, Maximal Inspiratory Pressure; MEP, Maximal Expiratory Pressure; FVC, Forced Vital Capacity; FEV1, Forced Expiratory Volume in 1 s; DE, Diaphragmatic Excursion; DT, Diaphragmatic Thickness; TUG, Timed Up and Go test; BBS, Berg Balance Scale; Mini-BESTest, Mini Balance Evaluation System Test; TIS, Trunk Impairment Scale; r, Correlation Coefficient. Bold type in tables indicates statistical significance.

### 3.3 Correlation between balance and postural control with respiratory and ultrasound outcomes

A high positive correlation was observed between BBS and MIP, the percentage of FEV1, and diaphragmatic excursion. The BBS also had a very high positive relationship with MEP and percentage of FVC. The Mini-BESTest showed a high positive correlation with MEP, percentage of FVC, and percentage of FEV1. Furthermore, a high positive correlation was found between TIS and MIP and between TIS and diaphragmatic excursion. TIS also correlated positively and highly with percentage of FVC, percentage of FEV1, and MEP. No correlations were observed between TUG and respiratory or ultrasound variables, except for the FEV1/FVC ratio with a high correlation (Table 4).

**Table 4:** Results of correlations between balance variables and respiratory and ultrasound outcomes.

	*MIP* *r (p)*	MEP *r (p)*	FVC *r (p)*	FEV1 *r (p)*	FEV1/FVC *r (p)*	DE *r (p)*	DT *r (p)*
*TUG*	−0.143 (*p* = 0.760)	−0.408 (*p* = 0.364)	−0.679 (*p* = 0.094)	−0.214 (*p* = 0.645)	0.821 **(*p* = 0.023)**	−0.571 (*p* = 0.180)	0.000 (*p* = 1.000)
*BBS*	0.589 **(*p* = 0.013)**	0.783 **(*p* = 0.000)**	0.709 **(*p* = 0.001)**	0.615 **(*p* = 0.009)**	−0.245 **(*p* = 0.344)**	0.591 **(*p* = 0.012)**	0.176 (*p* = 0.499)
*Mini-BESTest*	0.386 (*p* = 0.126)	0.686 **(*p* = 0.002)**	0.596 **(*p* = 0.012)**	0.518 **(*p* = 0.033)**	−0.088 (*p* = 0.737)	0.481 (*p* = 0.051)	0.059 (*p* = 0.822)
*TIS*	0.630 **(*p* = 0.007)**	0.849 **(*p* = 0.000)**	0.752 **(*p* = 0.000)**	0.711 **(*p* = 0.001)**	−0.144 (*p* = 0.580)	0.615 **(*p* = 0.009)**	0.250 (*p* = 0.333)

MIP, Maximal Inspiratory Pressure; MEP, Maximal Expiratory Pressure; FVC, Forced Vital Capacity; FEV1, Forced Expiratory Volume in 1 s; ED, Diaphragmatic Excursion; GD, Diaphragmatic Thickness; TUG, Timed Up and Go test; BBS, Berg Balance Scale; Mini-BESTest, Mini Balance Evaluation System Test; TIS, Trunk Impairment Scale; r, Correlation Coefficient. Bold type in tables indicates statistical significance.

### 3.4 Correlations between fatigue and respiratory and ultrasound outcomes

Correlations were conducted between the Borg Dyspnea Scale and the VAS-F with the parameters of FVC, FEV1, FEV1/FVC ratio, MIP, and MEP, as well as with diaphragmatic thickness and diaphragmatic excursion during forced inspiration. None of them were statistically significant, except for the Borg Dyspnea Scale with the FEV1/FVC ratio, which showed a very high positive correlation (Table 5).

**Table 5:** Results of the correlations between fatigue and respiratory and ultrasound outcomes.

	*MIP* *r (p)*	MEP *r (p)*	FVC *r (p)*	FEV1 *r (p)*	FEV1/FVC *r (p)*	DE *r (p)*	DT *r (p)*
*Borg (fatigue during an activity)*	−0.256 (*p* = 0.579)	−0.368 (*p* = 0.417)	−0.591 (*p* = 0.162)	0.118 (*p* = 0.801)	0.867 **(*p* = 0.012)**	0.650 (*p* = 0.114)	0.000 (*p* = 1.000)
*VAS-F (general fatigue)*	0.056 (*p* = 0.831)	−0.362 (*p* = 0.154)	−0.404 (*p* = 0.108)	−0.328 (*p* = 0.199)	0.365 (*p* = 0.150)	−0.442 (*p* = 0.076)	0.160 (*p* = 0.54)

MIP, Maximal Inspiratory Pressure; MEP, Maximal Expiratory Pressure; FVC, Forced Vital Capacity; FEV1, Forced Expiratory Volume in 1 s; DE, Diaphragmatic Excursion; DT, Diaphragmatic Thickness; VAS-F, Visual Analog Scale for Fatigue; r, Correlation Coefficient. Bold type in tables indicates statistical significance.

### 3.5 Correlations between fatigue and balance and postural control

A very high negative correlation was observed between the Borg Dyspnea Scale and the BBS, as well as between the Borg Dyspnea Scale and the Mini-BESTest. It was also noted that, in the group of persons with MS, there is a very high positive correlation between the TUG and the Borg Dyspnea Scale and an almost perfect correlation between the TUG and the VAS-F (Table 6).

**Table 6:** Results of the correlations between fatigue and balance and postural control.

	*TUG* *r (p)*	*BBS* *r (p)*	*Mini-BESTest r (p)*	*TIS* *r (p)*
*Borg (fatigue during an activity)*	0.867 **(*p* = 0.012)**	−0.768 **(*p* = 0.044)**	−0.775 **(*p* = 0.041)**	−0.470 (*p* = 0.287)
*VAS-F (general fatigue)*	0.927 **(*p* = 0.003)**	0.207 (*p* = 0.424)	−0.447 (*p* = 0.072)	−0.207 (*p* = 0.424)

TUG, Timed Up and Go test; BBS, Berg Balance Scale; Mini-BESTest, Balance Evaluation System Test; TIS, Trunk Impairment Scale; VAS-F, Visual Analog Scale for fatigue; r, correlation coefficient. Bold type in tables indicates statistical significance.

## 4 Discussion

Based on the results obtained, no statistically significant differences were observed in diaphragmatic thickness measured by ultrasound between persons with MS and healthy subjects. Similarly, no significant differences were found in the FEV1/FVC ratio. However, statistically significant differences were identified between the two groups in diaphragmatic excursion, MIP, MEP, FVC, and FEV1. These findings reaffirm the hypothesis positing respiratory functional impairments in persons with MS. One plausible explanation may involve motor airway disturbances, as proposed by Westerdahl et al. (46) rather than morphological factors like diaphragmatic thickness (47). The absence of differences in the FEV1/FVC ratio may be attributed to the fact that persons with MS may exhibit a restrictive pattern. Where this index remains within the normal range (48).

In the correlation analysis between the various variables analyzed in persons with MS, our results show that respiratory variables, such as inspiratory and expiratory respiratory pressures (MIP, MEP), exhibit a strong correlation with variables related to balance and postural control in persons with MS. Likewise, pulmonary function measured in terms of spirometric volumes (FVC, FEV1) and diaphragmatic excursion, as measured by ultrasound, also showed a strong correlation with variables related to postural control and balance. Previous studies conducted in other pathologies have demonstrated this relationship. In stroke patients, a training regimen incorporating inspiratory muscle exercise and core training improved gait balance (23). In patients with Parkinson’s disease, it was concluded that diaphragmatic functionality and postural control were interrelated (24). In healthy subjects, diaphragm thickness was associated with static balance (17). Good postural control improves respiratory capacities, and conversely, optimal respiratory function enhances postural control (17). On the other hand, if postural control is compromised. The diaphragm may not participate as effectively in ventilatory mechanics (21). Confirming these findings in persons with MS opens the door to designing comprehensive therapeutic strategies that incorporate core strengthening.

In the results of this study, a significant relationship was observed between Borg fatigue variables and the status of balance systems, and postural control, in persons with MS. However, overall fatigue did not correlate with respiratory capacity. These findings align with previous research that has demonstrated the relationship between postural control and fatigue (21, 49–51). Van Emmerik et al. (51) reported that persons with MS with lower limb weakness. Motor control issues, stability problems, and postural control issues exhibited increased postural activity, which correlated with fatigue. Similarly, Santinelli et al. (50) observed that in individuals with minimally affected MS, fatigue manifested earlier during activities that demanded a high level of postural control compared to healthy subjects. Callesen et al. (52) described how strength and trunk stability training improved perceived fatigue in persons with MS. In a related vein, Hebert et al. (49) found an association between fatigue and balance and suggested that this might be explained by central sensory integration problems.

The fact that we did not find a positive correlation between fatigue and respiratory variables in this study contradicts the findings of Balkan et al. (31) and Ray et al. (32), who reported a relationship between the presence of fatigue in persons with MS and a decrease in respiratory capacities. Furthermore, our results differ from the conclusions of Martín-Valero et al. (30) and Martín-Sánchez et al. (53), who identified a positive correlation between respiratory function training and an improvement in perceived fatigue in persons with MS (30, 53).

It has been observed that persons with lower limb weakness, motor control and stability issues, and cognitive problems exhibit increased postural activity correlated with fatigue (51). Additionally, in individuals with minimally affected MS, fatigue manifests earlier during activities requiring a high level of cognitive processing compared to healthy subjects (50). Our findings contrast with studies that have found a positive correlation between respiratory function training and perceived fatigue improvement in persons with MS (53). These conclusions underscore the complexity of fatigue as a phenomenon in persons with MS and the therapeutic approaches required. The relationship between mobility, diaphragmatic functionality, and both balance and respiratory functionality suggests the possibility that therapeutic interventions aimed at improving diaphragmatic function, broadly speaking, may influence fatigue and postural control.

This study is not without limitations. Dividing subjects into different levels of EDSS and different types of MS would allow for studying differences among persons with MS at various levels of functionality. A larger sample would be needed to extrapolate the conclusions to different MS groups.

In conclusion, the results of this study indicate that there are no morphological differences in the core between persons with MS and healthy subjects. However, differences in respiratory patterns and function can be observed. Furthermore, in persons with MS, a significant relationship exists between balance and respiratory function, as well as between balance and fatigue. Nevertheless, fatigue does not correlate with respiratory variables. Consequently, interventions aimed at enhancing balance and postural control are likely to have a positive impact on perceived fatigue and respiratory function. Likewise, improvements in respiratory function may positively influence postural control. Further research is needed to delve deeper into these relationships and their clinical implications.

## Data availability statement

The original contributions presented in the study are included in the article/supplementary material, further inquiries can be directed to the corresponding author.

## Ethics statement

The studies involving humans were approved by HOSPITAL CLINICO SAN CARLOS C/ Professor Martín Lagos, s/n. 28,040 Madrid. The studies were conducted in accordance with the local legislation and institutional requirements. The participants provided their written informed consent to participate in this study. Written informed consent was obtained from the individual(s) for the publication of any potentially identifiable images or data included in this article.

## Author contributions

RS-R: Conceptualization, Data curation, Investigation, Methodology, Formal analysis, Writing – original draft, Writing – review & editing. MdlPSF: Conceptualization, Data curation, Investigation, Methodology, Formal analysis, Writing – original draft, Writing – review & editing. MDS-R: Conceptualization, Data curation, Investigation, Methodology, Formal analysis, Writing – original draft, Writing – review & editing. IS-E: Conceptualization, Data curation, Investigation, Methodology, Formal analysis, Writing – original draft, Writing – review & editing. MG-A: Conceptualization, Data curation, Investigation, Methodology, Formal analysis, Writing – original draft, Writing – review & editing. CE-B: Conceptualization, Data curation, Investigation, Methodology, Formal analysis, Writing – original draft, Writing – review & editing.
